# An Inconspicuous Offender: Apixaban-Induced Anticoagulant-Related Nephropathy

**DOI:** 10.7759/cureus.44672

**Published:** 2023-09-04

**Authors:** Dylan J Shaw, Stephanie Kaiser, Alexander Kong, Shivam Joshi

**Affiliations:** 1 Department of Medicine, University of Central Florida College of Medicine, Orlando, USA; 2 Pharmacy, Orlando Veterans Affairs Healthcare System, Orlando, USA; 3 Cardiology, Orlando Veterans Affairs Healthcare System, Orlando, USA; 4 Cardiology, Orlando Health Heart and Vascular Institute, Orlando, USA; 5 Nephrology, Orlando Veterans Affairs Healthcare System, Orlando, USA; 6 Department of Medicine, New York University Grossman School of Medicine, New York, USA

**Keywords:** anticoagulant-related nephropathy, nephropathy, direct oral anticoagulant (doac), kidney injury, apixaban

## Abstract

Direct oral anticoagulants (DOACs) have shifted the landscape of anticoagulation over the past decade, becoming a frequently used pharmaceutical agent. The increased use of DOACs for long-term anticoagulation has led to a rise in reported anticoagulant-related adverse reactions, such as anticoagulant-related nephropathy (ARN). The occurrence of ARN is well reported with warfarin; however, there are few cases of ARN reported with DOAC use. We report the case of an elderly man with coronary artery disease and hypertension who was initiated on apixaban for atrial fibrillation three years prior to presentation but developed rapid renal decline over the six months prior to presentation. The estimated glomerular filtration rate (eGFR) had decreased precipitously from 48 mL/min/1.73 m^2^ to 19 mL/min/1.73 m^2^ with a concurrent drop in hemoglobin in the setting of persistent microscopic hematuria. A renal biopsy showed red blood cell casts consistent with glomerular hematuria, despite no crescents or signs of other forms of glomerulonephritis. The patient’s renal function ceased to deteriorate and had a 35% recovery (serum creatinine 2.6 mg/dL, eGFR 25 mL/min/1.73 m^2^) after the discontinuation of apixaban and conversion to rivaroxaban without the use of corticosteroids. The patient reported at follow-up that he discontinued rivaroxaban four days after initiation on his own accord due to extrarenal bleeding. Our case highlights the importance of prompt recognition and treatment of the underreported but potentially significant incidence of ARN with apixaban in a patient with an otherwise unexplained kidney injury.

## Introduction

The emergence of direct oral anticoagulants (DOACs) has reshaped the landscape of anticoagulation over the past decade, with proven noninferiority and, in some indications, superiority over warfarin in terms of safety and efficacy in a variety of treatment scenarios. Between 2011 and 2020, DOAC use increased significantly from 4.7% to 47.9% in patients with atrial fibrillation, while warfarin use decreased from 52.4% to 17.7% [1]. The recommendation of DOAC therapy over that of warfarin within major guidelines is not only based upon improved safety and an equal or superior efficacy profile but also because of increased convenience with regard to monitoring and dietary restrictions [2-9]. Despite a better safety profile, there appears to be growing concern with the incidence of anticoagulant-related nephropathy (ARN) reported in the literature among patients on DOAC therapy. Although there are only a small number of case reports at this time, dabigatran has the most reported cases of this phenomenon compared with the other DOACs, while apixaban and edoxaban have the least [10].

We present the case of biopsy-confirmed ARN while on apixaban for approximately three years, with an onset of kidney injury noted after approximately 14 months of therapy. Anecdotally, the incidence of ARN with DOACs is likely underreported, as it appears to be a common finding in renal pathology examinations. Nonetheless, to our knowledge, we report the second case of ARN with apixaban [11].

## Case presentation

The patient was a 77-year-old Caucasian male with a past medical history of hyperlipidemia, hypertension, coronary artery disease with two-vessel coronary artery bypass graft surgery three years prior, right internal carotid artery stenosis with carotid endarterectomy two and a half years prior, paroxysmal atrial fibrillation (CHA_2_DS_2_-VASc score = 4; HAS-BLED score = 3), chronic kidney disease (CKD) stage 2, and dry macular degeneration. The patient denied any history of excessive bleeding while on anticoagulation at presentation but revealed at follow-up that he had extrarenal bleeding from his eyes, mouth, and anus.

**Figure 1:**
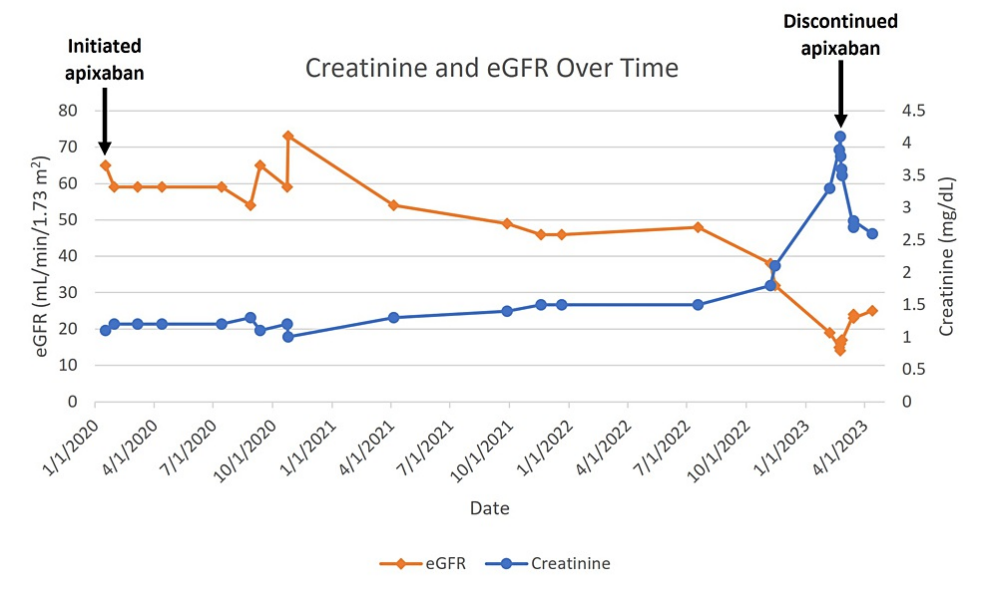
Changes in serum creatinine and estimated glomerular filtration rate (eGFR calculated by CKD-EPI equation) over time EGFR: glomerular filtration rate; CKD-EPI: chronic kidney disease epidemiology collaboration

**Figure 2:**
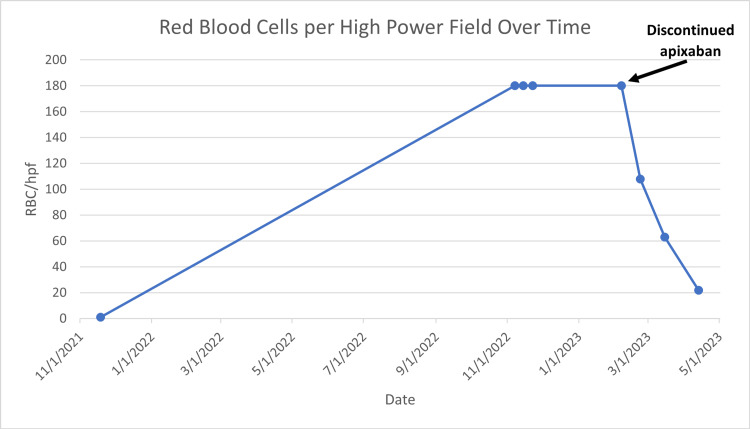
Changes in the number of red blood cells (RBC) per high power field (hpf) in urine over time

The patient presented to the nephrology clinic after being referred for evaluation of proteinuric CKD. The patient’s estimated glomerular filtration rate (eGFR) had been slowly declining for 22 months, with a rapid decrease from 48 mL/min/1.73 m2 (serum creatinine 1.5 mg/dL) to 19 mL/min/1.73 m2 (serum creatinine 3.3 mg/dL) over the course of six months (Figure 1). Additionally, the patient’s hemoglobin had decreased during the three months prior with persistent microscopic hematuria (Figure 2). Further non-invasive workup included a 1:40 antinuclear antibody (ANA) titer, negative antineutrophil cytoplasmic antibodies (ANCA), negative anti-glomerular basement membrane (GBM) antibody, negative hepatitis B surface antibody and core antibody, negative hepatitis C antibody, normal C3 and C4 complement, normal A1c, and negative HIV antigen and antibody. Given the negative results, a renal biopsy was recommended for further evaluation of hematuria and renal function decline. His home medications included apixaban (5 mg twice daily), aspirin (81 mg once daily), cholecalciferol (50 μg once daily), metoprolol succinate (25 mg once daily), rosuvastatin (20 mg once daily), and a multivitamin (twice daily). Additionally, upon interview, the patient admitted to taking non-prescription oral supplements, including adaptogens, nattokinase serrapeptase, seaweed powder extract, vitamin C, and other "mineral powders" that he did not know the names of for the past two to three months.

On hospital admission two weeks later, the patient was asymptomatic with a physical exam remarkable for bilateral lower extremity edema, obese body habitus (116.57 kg), and irregular rhythm with a blood pressure of 134/80 and a heart rate of 83 beats per minute. The patient’s eGFR had decreased to 14 mL/min/1.73 m^2^ (creatinine 4.1 mg/dL), and labs were remarkable for a blood urea nitrogen of 45 mg/dL, a hemoglobin of 9.5 g/dL, urine protein-creatinine ratio of 601 mg/g, and 180 red blood cells per high power field.

**Figure 3:**
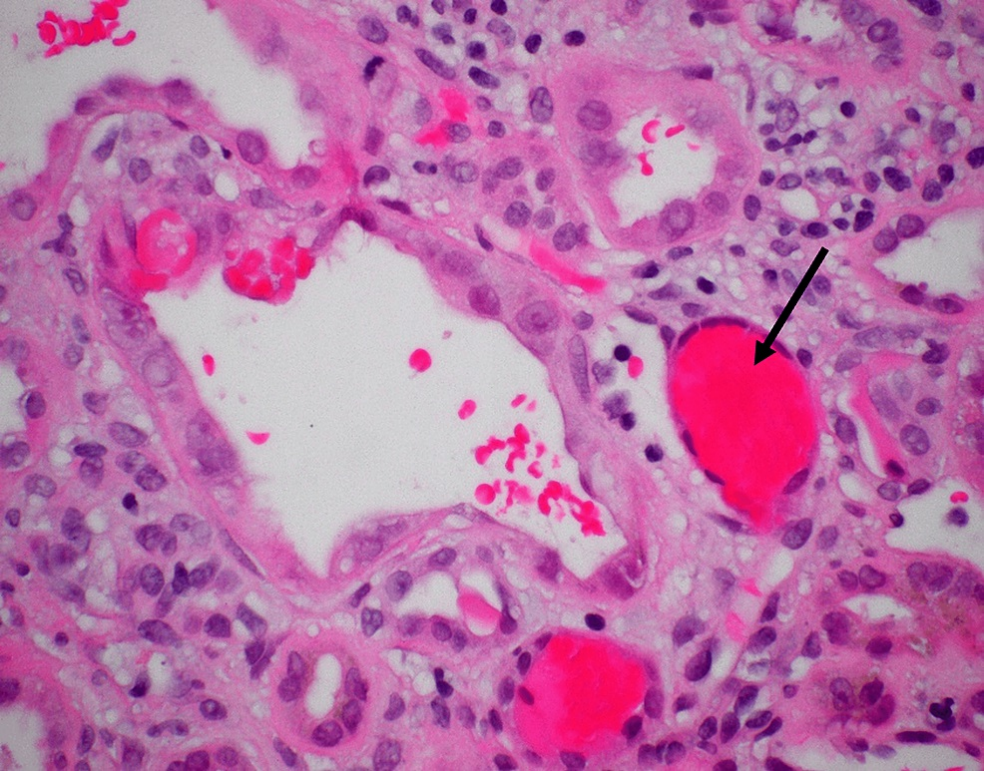
Red blood cell casts in tubular lumina

**Figure 4:**
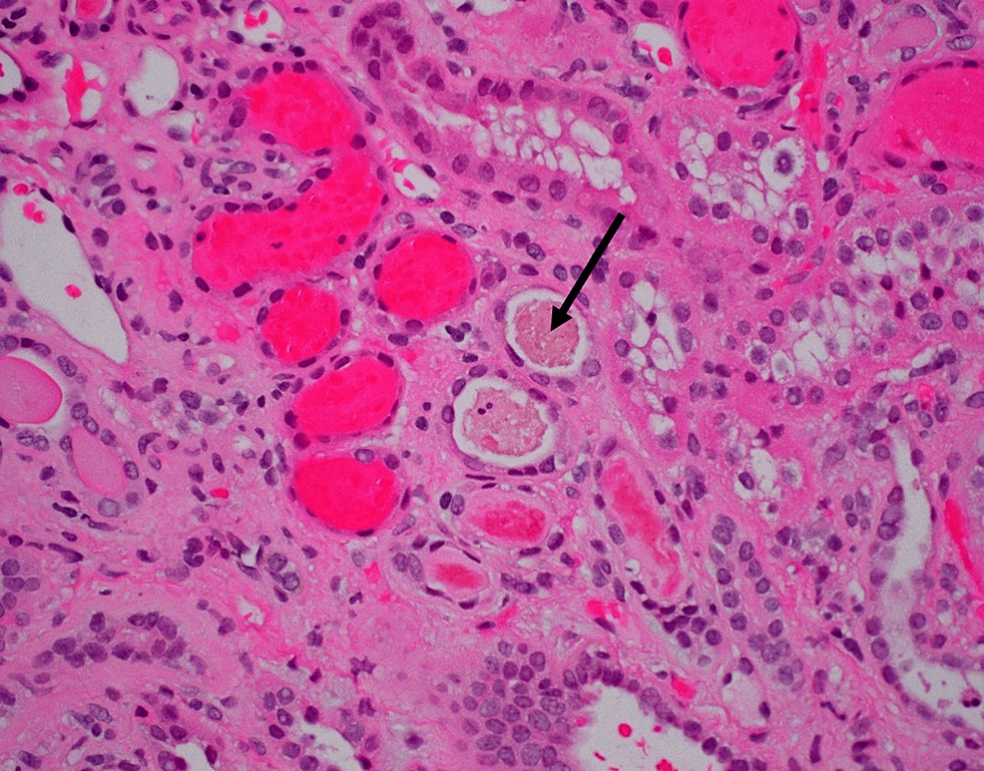
Erythrophagocytosis by tubular epithelial cells

Light microscopy from the renal biopsy revealed acute tubular injury with many red blood cell casts, marked arteriosclerosis, moderate interstitial fibrosis, and tubular atrophy (40%) (Figures 3, 4). No crescents or signs of other forms of glomerulonephritis were present. Immunofluorescence microscopy showed trace staining for IgA, C3, Kappa and Lambda in a granular pattern in the mesangium. Tubular casts stained positively for IgA (1+), Kappa (1+), and Lambda (1+). Electron microscopy revealed glomerular basement membranes that were wrinkled in an ischemic pattern, as well as subtotal effacement of podocyte foot processes. Occasional small electron-dense deposits were present in mesangial regions.

Upon consultation, cardiology recommended the initiation of an alternative DOAC, rivaroxaban, given the increased incidence of ARN with dabigatran and warfarin. It was also recommended that the patient be evaluated for elective percutaneous closure of the left atrial appendage (LAA) to avoid long-term exposure to anticoagulation. Additionally, the patient was instructed to discontinue all non-prescribed vitamin supplements and resume low-dose aspirin.

After discontinuation of apixaban, the patient’s creatinine improved from 4.1 mg/dL to 2.6 mg/dL in the following seven weeks (Figure 1). The patient’s hemoglobin remained low but stable, with follow-up testing showing a hemoglobin concentration of 9.0 g/dL at three weeks following apixaban discontinuation.

At further follow-up, the patient reported for the first time that he had extrarenal bleeding (eyes, mouth, and anus) with apixaban that had continued after conversion to rivaroxaban. The patient reported that he had made the decision to self-discontinue rivaroxaban and did not desire further anticoagulation. The patient maintained his position of not restarting a DOAC despite being counseled on the benefits of it because his extrarenal bleeding had stopped after stopping the DOAC. The patient was subsequently referred to hematology for further workup of his bleeding after using two DOACs.

## Discussion

Utilization of DOAC therapy, specifically with atrial fibrillation, has increased significantly after proven efficacy and improved safety over warfarin in landmark trials [2-9]. Despite an improved safety profile, there is a growing concern about the occurrence of ARN with DOAC use. This concern appears to be underreported in the literature. To date, cases of ARN have been most frequently reported with dabigatran use, with only one previous case reported for apixaban and edoxaban [10]. Upon review of the FDA Adverse Event Reporting System (FAERS) Public Dashboard, a total of 65 cases were reported for ARN use from 2020 to the present date, with no events reported for apixaban. Of note, 49% (n=32) of the cases occurred with warfarin; 14% (n=9) of the documented cases occurred with dabigatran; 11% (n = 7) of the events occurred with rivaroxaban; and 8% (n=5) of the cases occurred with edoxaban. Interestingly, two events (3%) were reported for aspirin. Using more general search terms of "acute kidney injury," "renal impairment," and "renal failure" for each drug increased the results of adverse events substantially, with apixaban having over 1,000 reported cases over the same timeframe of 3.5 years. This calls into question how many cases of ARN are wrongly identified as acute kidney injury or renal impairment.

The proposed mechanism of ARN is still not fully understood, but nephron damage appears to have an obstructive etiology. The development of casts from red blood cells creates obstruction, further damaging the nephrons, and continued lysis of red blood cells leads to the accumulation of heme-containing molecules and catalytic iron, which creates detrimental hydroxyl radicals [10]. Common risk factors associated with ARN development include overdosing on anticoagulation, concomitant use of interacting medications, CKD, advanced age, diabetes, hypertension, and heart failure [12-14]. The incidence of ARN with CKD has been speculated to be as high as 33% in patients receiving warfarin therapy, but the incidence with DOAC therapy is also unknown [15]. This is particularly concerning as apixaban’s use in patients with advanced kidney disease continues to grow since the publication of the Apixaban for Reduction in Stroke and Other Thromboembolic Events in Atrial Fibrillation (ARISTOTLE) trial [2], where people were only excluded if their creatinine clearance was less than 25 mL/min. Recently, the FDA approved apixaban’s use in hemodialysis patients, given the results of the RENAL-AF trial [16]. Even though CKD is a known risk factor for the development of ARN, the incidence of ARN with warfarin therapy has been reported as high as 16.5% in non-chronic kidney disease with a presentation of acute kidney injury, emphasizing the importance of prompt recognition of ARN in all levels of renal disease receiving DOAC therapy as that incidence rate remains unknown [15]. However, it should be noted that renal biopsies were not performed in these patients. In a retrospective meta-analysis of 24 cases, only 40% of cases had renal recovery after anticoagulation cessation, with the majority of patients having no recovery in their renal function [10]. In this patient’s case, although his renal function has recovered to a small degree after DOAC self-discontinuation, it is difficult to conclude if the patient will continue to recover at this time.

Our patient was on apixaban for atrial fibrillation for three years prior to developing rapidly advancing renal disease in the setting of microscopic hematuria. Following confirmation with a renal biopsy, the diagnosis of ARN was made, making this the second reported case of apixaban-induced ARN [11]. An important aspect of this case is the concomitant use of aspirin and apixaban, which possibly contributed to the development of ARN. In 2018, a case report was published regarding the development of ARN with the use of dual-antiplatelet therapy (DAPT), suggesting that the mechanism for the development of ARN extends beyond that of anticoagulation therapy [17]. The combination of antiplatelets and anticoagulants is generally not recommended beyond 12 months after an acute coronary event or coronary intervention unless the bleeding risk is low [18]. The addition of an antiplatelet agent to an anticoagulant can increase the bleeding risk by more than 20% [18]. In this case report, the patient had chronic kidney disease, which is an independent risk factor for bleeding; therefore, his bleeding risk was increased significantly with the addition of aspirin to apixaban, which is reflected in the patient’s HAS-BLED score of 3.

Lastly, it is important to note that the patient was on several over-the-counter herbal supplements and vitamins. Although the contribution of these supplements to the development of ARN and acute kidney injury is unknown, it is important to emphasize the risk of adverse effects of herbal supplements, particularly when combined with prescription medications. Herbal supplements and over-the-counter products can increase the risk of developing acute kidney injury, cause alterations in anticoagulation pharmacokinetics, and/or interact pharmacodynamically by increasing bleeding risk independently, as is the case with nonsteroidal anti-inflammatory (NSAID) drugs, flaxseed, garlic, and vitamin E. Therefore, thorough medication reconciliation and evaluation of over-the-counter products are essential when assessing contributing factors for bleeding risk and ARN development.

Despite the growing awareness of ARN with DOAC therapy, the treatment for ARN is not well established. By evaluating published data, it is recommended to temporarily discontinue the offending agent with supportive care when ARN is diagnosed [19]. The treatment of ARN with DOAC therapy remains unknown at this time [19]. The use of corticosteroids has been seen in several case reports, but their mechanism in ARN is not fully understood. A Naranjo score was calculated to determine the probability of apixaban causing ARN in this patient, which equaled a score of 7, which was determined to be "a probable adverse drug reaction." Although corticosteroids were not used in our patient’s treatment plan, the patient’s renal function improved following apixaban discontinuation, indicating that apixaban was the most likely causative agent for our patient’s kidney injury.

## Conclusions

Since ARN with DOACs is an underreported and likely underdiagnosed phenomenon, prescribers may not be aware of the potential renal complications associated with DOAC use, particularly apixaban. Delayed recognition of ARN with DOACs can lead to further deterioration and long-term irreversible kidney injury if the DOAC is not discontinued or switched to another agent in a timely fashion. Given the widespread use of DOACs today, it is crucial that prescribers recognize the potential for ARN with DOACs, including apixaban, in patients with otherwise unexplained kidney injury.
